# Navigating diabetes-related immune epitope data: resources and tools provided by the Immune Epitope Database (IEDB)

**DOI:** 10.4172/1745-7580.1000063

**Published:** 2013

**Authors:** Kerrie Vaughan, Bjoern Peters, Roberto Mallone, Matthias von Herrath, Bart O. Roep, Alessandro Sette

**Affiliations:** 1 Vaccine Discovery, La Jolla Institute for Allergy and Immunology, La Jolla, CA; 2 INSERM, U1016, Cochin Institute, DeAR Lab Avenir, Saint Vincent de Paul Hospital, 82 Avenue Denfert Rochereau, 75674 Paris Cedex 14, France; 3 Developmental Immunology, La Jolla Institute for Allergy and Immunology, La Jolla, CA; 4 Department for Immunohematology and Blood Transfusion, Leiden University Medical Center, Leiden, Netherlands

## Abstract

**Background:**

The Immune Epitope Database (IEDB), originally focused on infectious diseases, was recently expanded to allergy, transplantation and autoimmunity diseases. Here we focus on diabetes, chosen as a prototype autoimmune disease. We utilize a combined tutorial and meta-analysis format, which demonstrates how common questions, related to diabetes epitopes can be answered.

**Results:**

A total of 409 references are captured in the IEDB describing >2,500 epitopes from diabetes associated antigens. The vast majority of data were derived from GAD, insulin, IA-2/PTPRN, IGRP, ZnT8, HSP, and ICA-1, and the experiments related to T cell epitopes and MHC binding far outnumbers B cell assays. We illustrate how to search by specific antigens, epitopes or host. Other examples include searching for tetramers or epitopes restricted by specific alleles or assays of interest, or searching based on the clinical status of the host.

**Conclusions:**

The inventory of all published diabetes epitope data facilitates its access for the scientific community. While the global collection of primary data from the literature reflects potential investigational biases present in the literature, the flexible search approach allows users to perform queries tailored to their preferences, including or excluding data as appropriate. Moreover, the analysis highlights knowledge gaps and identifies areas for future investigation.

## BACKGROUND

The present study illustrates how the Immune Epitope Database and Analysis Resource (IEDB), originally focused on infectious diseases [1], can be used to analyze epitope data related to type 1 diabetes (T1D) chosen as an exemplary disease. A combined tutorial and meta-analysis format demonstrates how common questions related to diabetes epitopes can be addressed using the IEDB and then also discusses the results of these queries.

T1D is characterized by autoimmune-mediated destruction of insulin-producing pancreatic beta cells, leading to insulin deficiency. CD4^+^ and CD8^+^ T cells have been implicated in T1D pathogenesis [2], and islet cell antibodies are diagnostic markers of disease. Mapping of diabetes associated auto antigens and epitopes enables the development of diagnostics to monitor beta cell autoimmunity and the design of novel immunoregulatory therapeutics [3, 4]. To facilitate achievement of these goals, existing epitope data should be made as easily accessible as possible to the scientific community.

The IEDB [5] (www.iedb.org) is a comprehensive repository of epitope data reported from the scientific literature. It includes antibody and T cells epitopes for infectious disease, allergy, autoimmunity and transplant-related disease. The IEDB is ‘assay centric,’ meaning that the specific experimental conditions underpinning the definition of the epitope are captured [6]. This provides immunological context to the data, identifying the host in which the epitope was defined, disease status or immunization procedures, the immunogen, the type (CD4^+^, IgG, etc.) of effector response measured, and the specific assay and antigen used. Journal articles are typically curated within 10 weeks of publication, making the content current with the published literature. Epitope data captured relates to either well defined minimal/optimal epitopes, or larger and less well-defined epitope-containing regions (20–50 residues), and “partial epitopes” including amino acid residues within epitopes identified as key for binding to immune receptors. Molecular structures that were tested for immune recognition but found to be unreactive are also reported as “negatives.”

Epitope reports can be subject to experimental biases and limitations, which may in turn result in the perpetuation of certain biases in the literature. The assay-centric nature of the IEDB was designed to support the scientific community, representing the data globally (analogous to Pub-Med) without arbitrary value judgments, but simultaneously providing a platform for selective inquiry and analysis.

The database design empowers users to easily include or exclude data as desired. For example, data from HLA transgenic mice can be excluded, by delimiting the query to only “*Homo sapiens*” as the host. Similarly a query can include only those data specific to an antigen of interest, excluding data related to potential unrelated epitopes, or select epitopes based on high or low MHC binding affinity (by specifying a range of IC50 values).

## RESULTS

### Inventory of diabetes-related data

A total of 409 references were classified as diabetes-related using automated classifiers combined with human expert review [7, 8]. These references describe 5,051 structures, including 2,537 peptides/molecular structures associated with at least one positive assay (epitopes) and 2,514 peptides/structures only associated with negative results (Table 1).

### Commonly reported diabetes-associated antigens

To identify diabetes associated antigens, we compiled the proteins which were the source of epitopes characterized in the references described above, removing proteins not derived from mammals, as they were sources as experimental controls (such as peptides from influenza).

**Figure 1:**
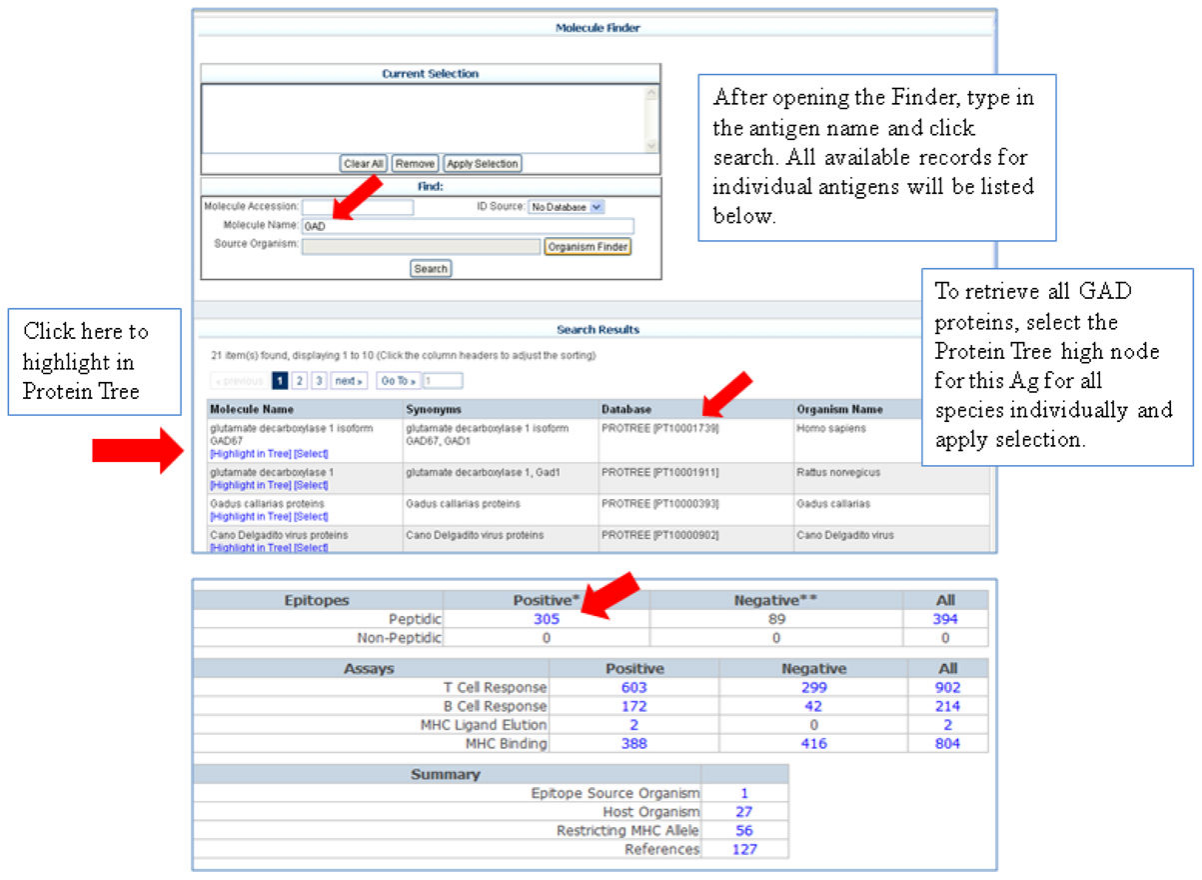

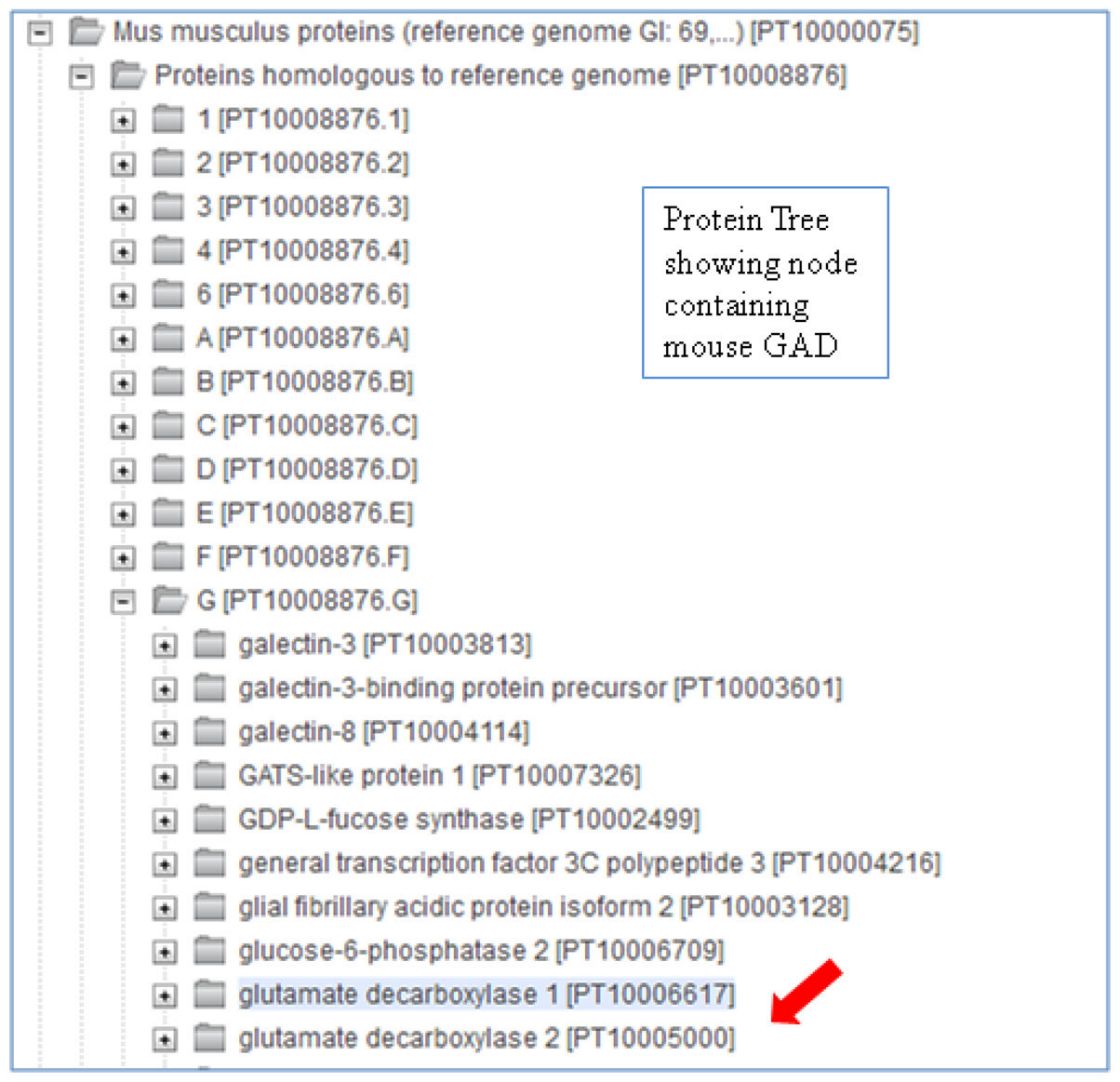
Figure 1A. Molecular Finder Entering ‘GAD’ in the Molecule Finder generates a list of available records for individual GAD entries (including all isoforms). Figure 1B. Protein Tree highlighting mouse GAD The protein Tree shows the two high nodes for mouse GAD1 and GAD2.

We next analyzed the nature of epitopes described for the most frequently reported antigens. For example, entering ‘GAD’ in the Molecule Finder we generate a list of available records for individual GAD entries (including all isoforms) (Figure 1A). The first entry in this list, ‘glutamate decarboxylase 1 isoform GAD67, PROTREE [PT10001739], Homo sapiens’ represents the high node on the Protein Tree for all human GAD proteins. The Protein Tree is a unique feature developed by the IEDB which organizes individually curated GenBank entries by sequence homology and thereby allows gathering of related records. To date, 305 structures associated with positive data (epitopes) are reported from a total 127 references. Human GAD epitopes were defined in several host species including, human, mouse and rabbit. T cell assays outnumber B cell assays by nearly 4 to 1. While a relatively high number of MHC binding records are available, only 2 positive ligand elution assays were reported. Records associated with related antigens (e.g. mouse GAD) can also be queried directly using the Protein Tree feature, accessible through the IEDB Molecule Finder [use ‘Highlight in Tree’]. Because the Protein Tree is organized by species, if different species (ex. mouse or rat) are considered, separate queries for each must be performed. Figure 1B also shows the Protein Tree highlighting mouse GAD.

Detailed data can be retrieved from the results summary table (Figure 1), by clicking on blue values that represent live links. Accordingly, it is possible to generate an epitope list, including sequence, antigen name (GenBank) and name of the organisms (NCBI) of origin. Similarly, details from specific assays, host organisms or a references list can be retrieved and also downloaded by clicking on the Excel icon at the top of the data table.

A total of 15 antigens reported in the IEDB account for 88% of the references and the near totality of the data (94%) (Table 2). GAD and insulin alone make up 62% of the curated assay data. The top seven antigens associated with more than 100 different curated assays (GAD, insulin, IA-2/PTPRN, ZnT8, HSP, IGRP and ICA-1 also known as ICA69), account for nearly 90% of the data. Additional antigens include MHC, glial fibrillary acidic protein (GFAP), serum albumin, islet amyloid polypeptide precursor (IAPP), insulin-like growth factor (IGF), TCR and chromogranin A. In this context, the serum albumin references relate to tolerance to cow milk in diabetes patients and induction of tolerance in general; similarly, several references describe the binding and immunogenicity or tolerogenicity of MHC and/or TCR derived peptides in the context of autoimmune diabetes (see below for tolerogenic peptide-based query). A few references describing non-peptidic antigens, such as lipopolysaccharide and lipooligosaccharides were also represented.

Table 2 summarizes positive assay data for the top diabetes-associated antigens. In most instances, T cell assays outnumbered B cell assays, except for IA-2 where we observed similar numbers of T and B cell assays. With respect to MHC binding, GAD had the most assays (514), followed by insulin (256), IA-2 (144), ICA-1 (136) and ZnT8 (66). Fewer MHC binding assays were reported for IGRP and HSP. Furthermore, few elution assays are reported for most antigens, with the exception of IA-2. Few data were reported to date for chromogranin-A, an antigen only recently identified as being associated with diabetes [9].

### Retrieving records related to specific epitopes

**Figure 2:**
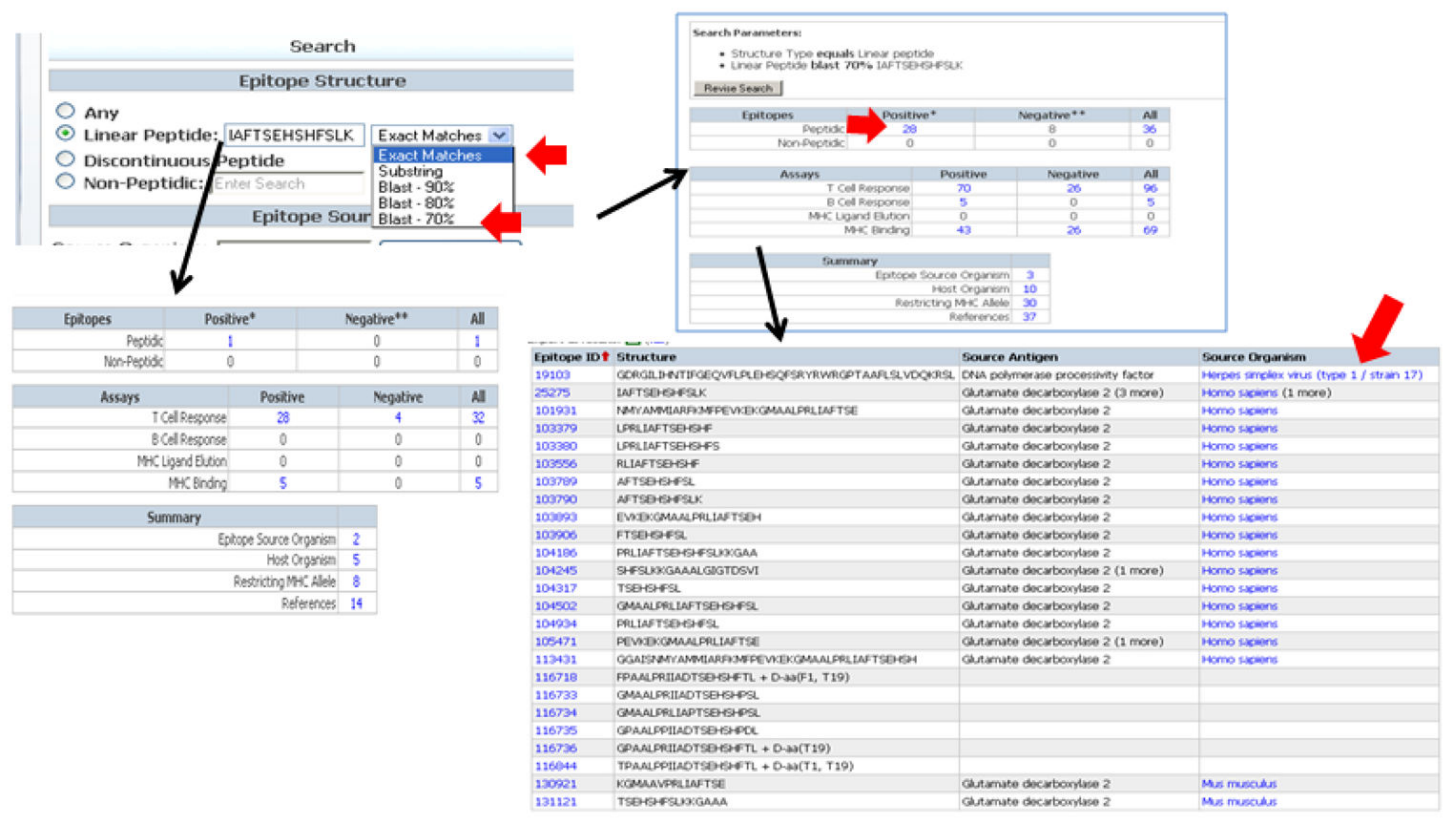

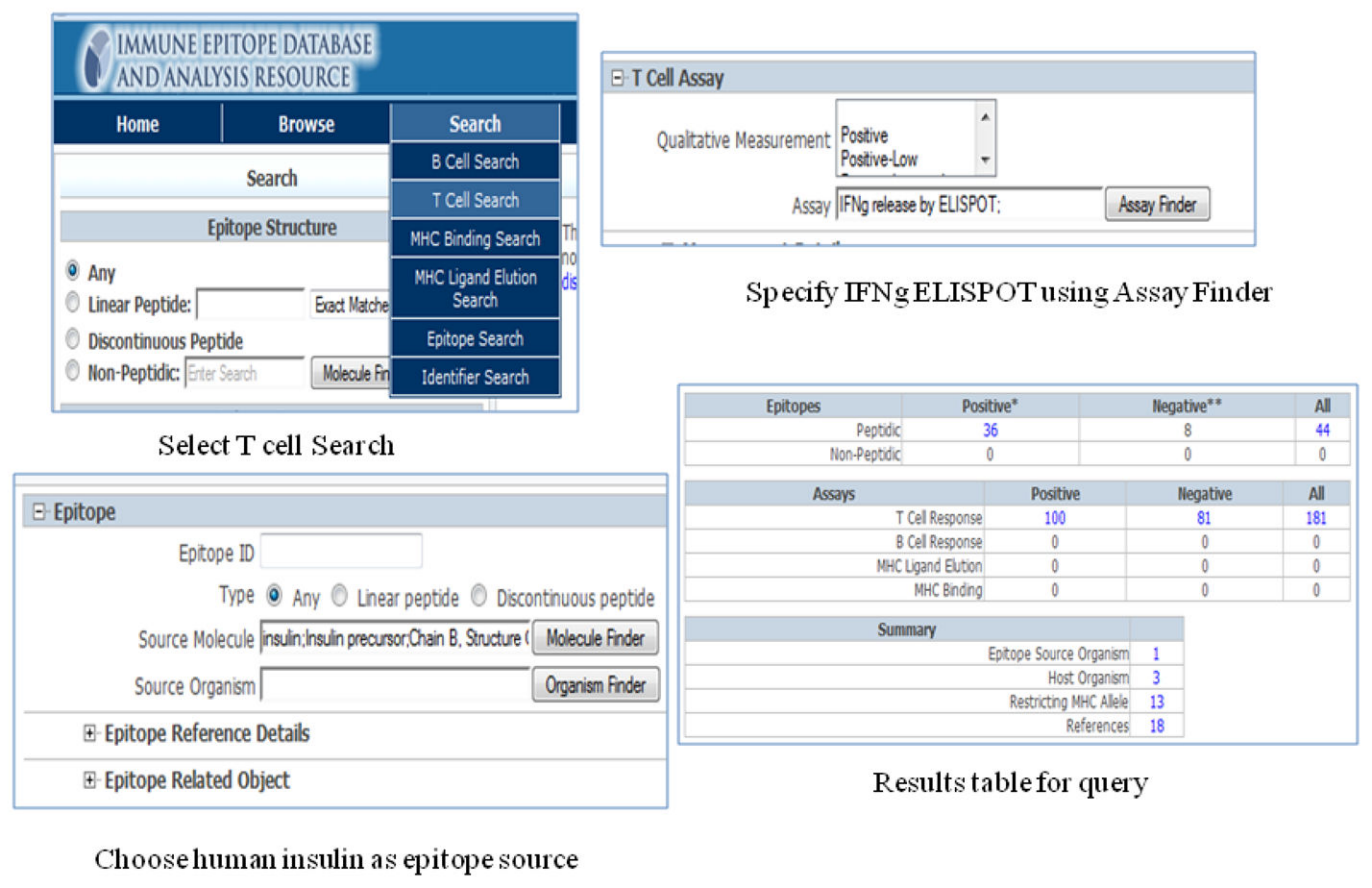

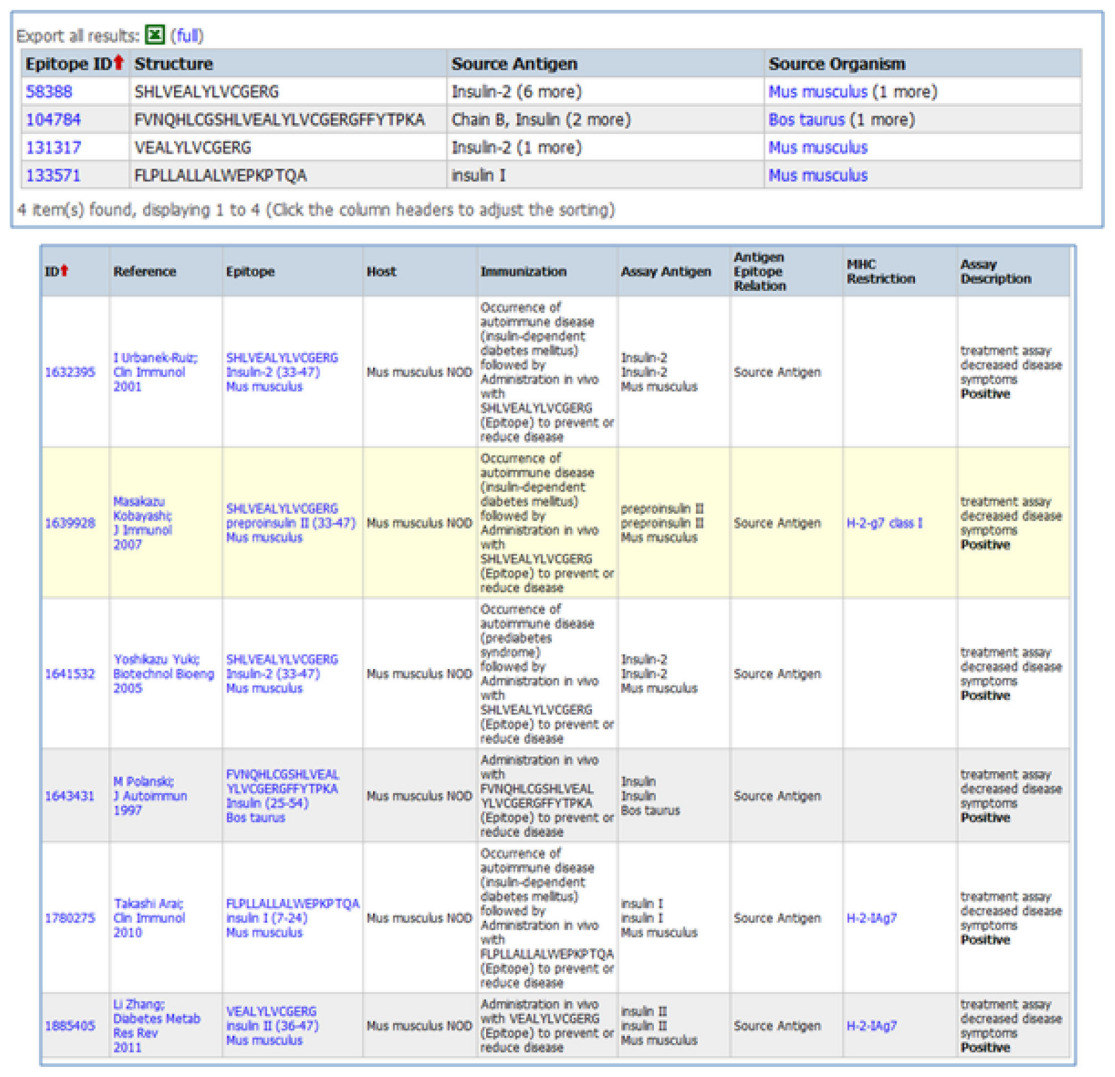
Figure 2A. Data for prominent class II GAD epitope (274–286) The ‘Linear peptide’ field on home page search interface was used to enter the GAD (274–286) sequence “IAFTSEHSHFSLK.” The sequence identity % of the BLAST search can be changed to find homologs of the entered sequence. Figure 2B. Advanced search for human insulin T cell epitopes defined using IFNγ ELISPOT From the pull down menu at the top of the home page, select ‘T cell Search.’ Next, under Epitope, the Molecule finder was used to select human insulin (sort on ‘Organism’ column to group all human proteins). Figure 2C. Tolerogenic T cell epitopes reported in NOD mice The advanced query was used to select insulin from all species, NOD mouse as host and T cell ‘Treatment assay.’

To exemplify how to retrieve information about a specific epitope, we choose the class II epitope GAD (274–286). In the ‘Linear peptide’ field on home page search interface we enter the GAD (274–286) sequence “IAFTSEHSHFSLK” (Figure 2A). This epitope was described in 14 references and tested in 28 different T cell assays. Clicking on the ‘8 Restricting MHC Allele’ in the table retrieves a list of alleles/serotypes tested, including DR4. MHC binding data are also available for this epitope. Clicking on the “5” MHC binding assays shows a summary of the different assays, including quantitative values (IC50 nM).

To obtain information about analogs and variants of an epitope, the sequence search can also be performed using different levels of sequence homology (Figure 2A). For example, a 70% sequence homology search retrieves 28 homologous epitopes, including synthetic analogs and a DNA polymerase epitope from herpes simplex virus. This feature can be used to identify epitopes of interest in the context of cross-reactivity (synthetic analogs and/or epitopes representing molecular mimics), potentially implicated in triggering autoimmunity through molecular mimicry [10, 11]. Homology searches can be further refined to take into account positional requirements derived from specific TCR and clonal requirements. [12–14].

The advanced search interface accessible from the pull-down menu along the top of the home page provides access to all the IEDB fields and can be used to search for additional details, such as assay type. For example, T cell epitopes defined for human insulin using IFNγ ELISPOT (Figure 2B) can be retrieved by selecting ‘human insulin’ using the Molecule Finder as the epitope source, and the Assay Finder to specify IFNγ ELISPOT. The query identifies 36 human insulin epitopes defined by 100 separate IFNγ ELISPOT assays from 18 references. This type of query can limit searches to a more stringent data subset (ex. assays representing immune correlates), or alternatively, to compare different data subsets (ex. human ELISPOT data versus mouse ELISPOT data).

Similarly the assay finder can be utilized to identify records defining tolerogenic peptides, defined as those peptides involved in the reduction (delayed onset) or treatment (decreased incidence) of disease *in vivo* following the administration of the epitope, or the adoptive transfer of epitope-specific effector cells. In some cases tolerogenic epitopes are defined using *in vitro* assays (ex. down -regulation of Ab response to HSP as hallmark of disease). As an example, the advanced query was used to select insulin from all species, NOD mouse as host and T cell ‘Treatment assay.’ The results show 4 epitopes, including the well known B9-23 peptide. Clicking on the ‘6’ positive assays show a summary of these records (Figure 2C).

**Figure 3:**
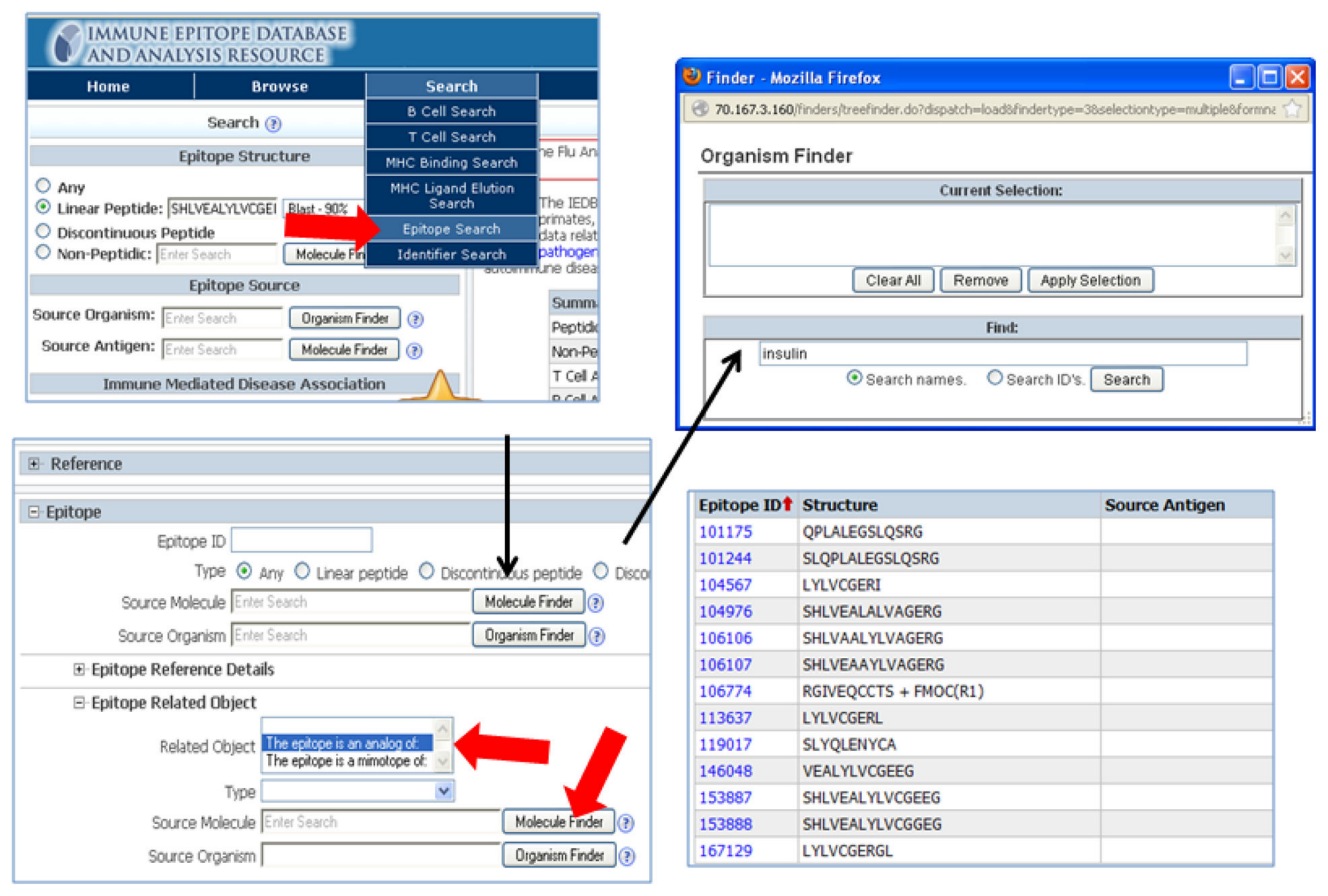
Analogs of insulin using advanced search A query to search for T cell analogs of insulin was performed. In the T cell Search under Epitope, the Epitope Related Object menu was expended by clicking the ‘+’ sign. Here, under Related Object, ‘The epitope is an analog of’ was highlighted and the Molecule Finder below was used to select insulin from all species.

Numerous studies analyzed analogs identified through peptide libraries or mimotopes. These are displayed in the query results as related to the host or source antigen, but shown in the results summary as peptides without source antigen or source organism. Figure 3 shows a query to search for T cell analogs of insulin. In the T cell Search under Epitope, the Epitope Related Object menu was expended by clicking the ‘+’ sign. Here, under Related Object, ‘The epitope is an analog of’ was highlighted and the Molecule Finder below was used to select insulin from all species.

### Searching for epitopes presented by specific alleles

Epitopes for which an MHC restriction has been determined or inferred are potentially useful for understanding the disease process, and can be employed as reagents or tools for diagnosis [15, 16]. The HLA class II loci DQA1, DQB1, and DRB1 have been associated with predisposition to T1D [17–21]. Similarly, the murine class II molecule IAg7 expressed in NOD mouse contributes to disease susceptibility, and the class II I-Ag7 β chain carries the same ‘diabetogenic’ amino acid residues found in the human DQB1*0302 allele associated with high risk for T1D development [22, 23]. Here, we exemplify three strategies associated with decreasing stringency: 1) epitopes for which validated tetramer reagents have been described, 2) epitopes with restriction defined in specific T cell assays 3) MHC binding data.

**Figure 4:**
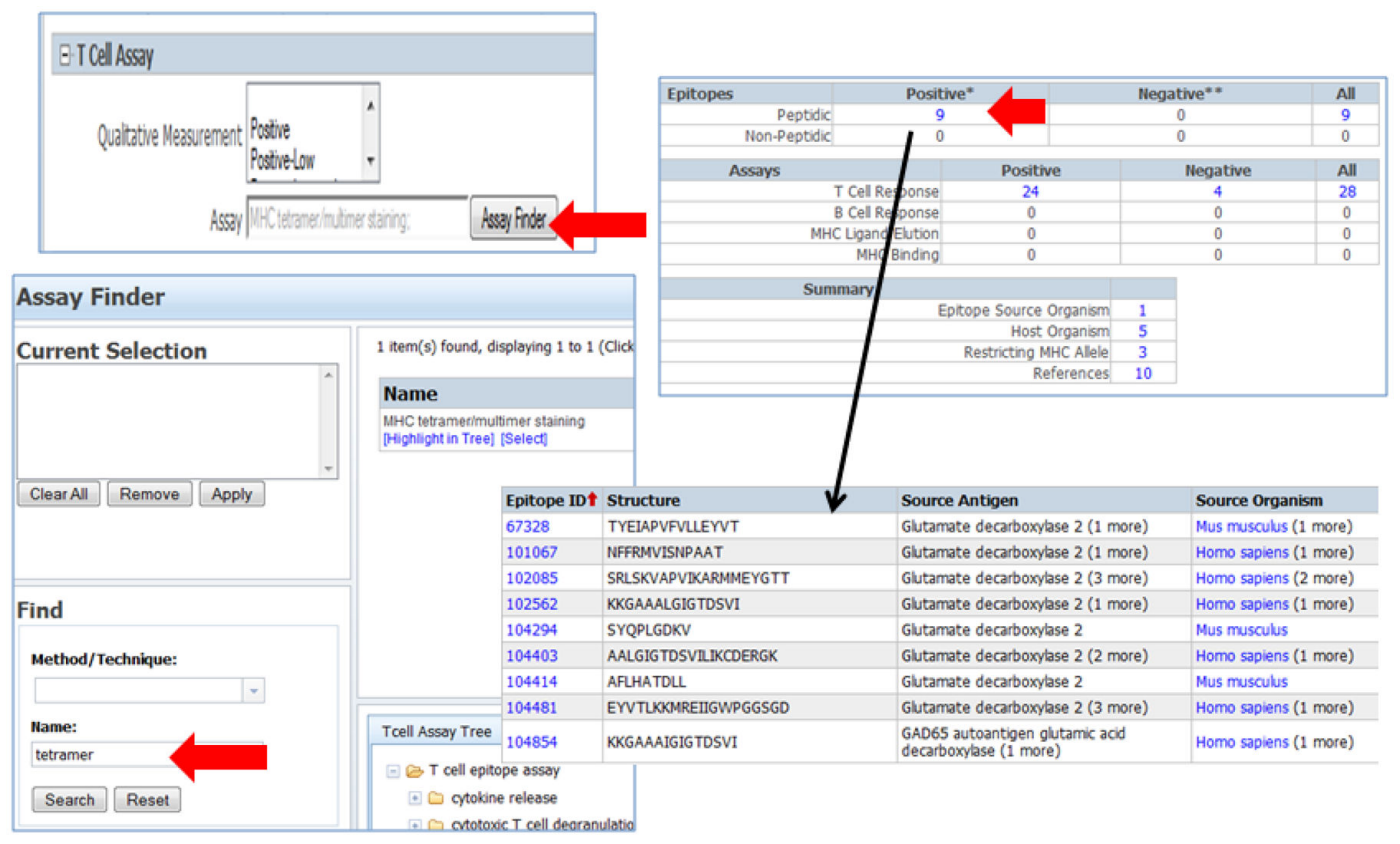

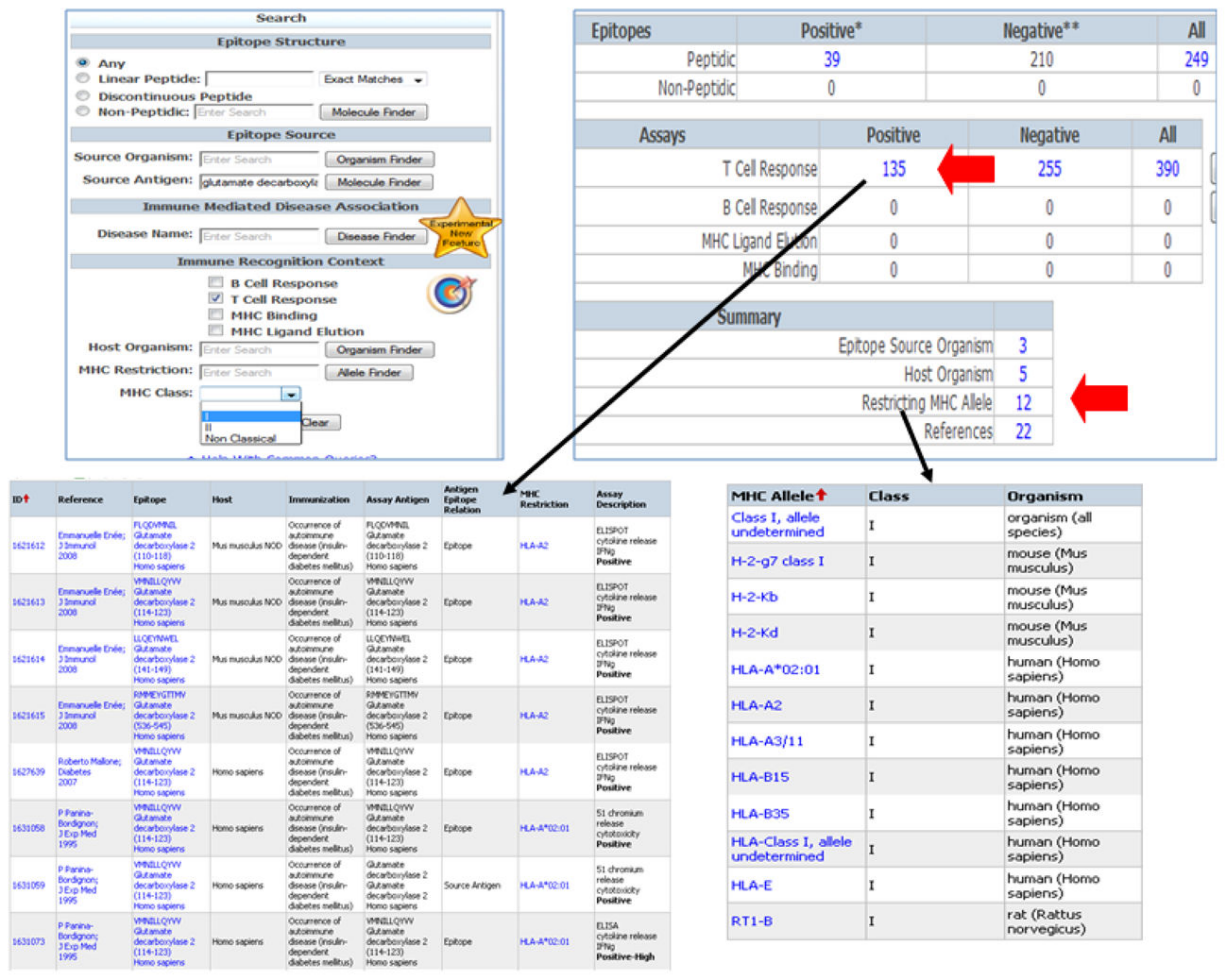

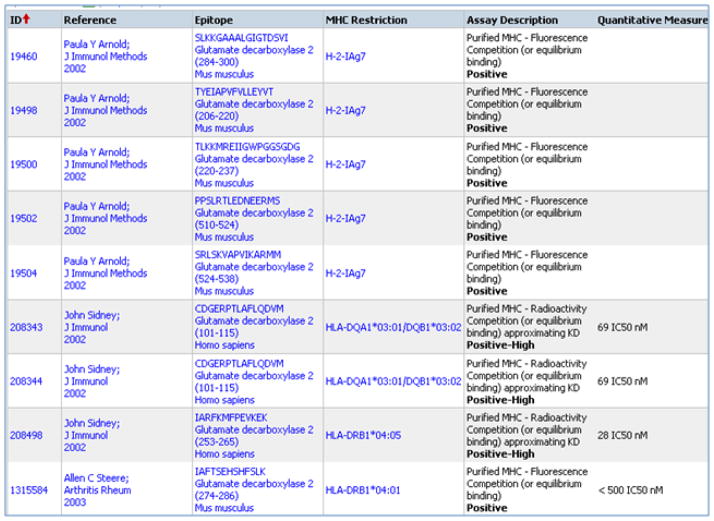
Figure 4A. Advanced search to select tetramer assays A) To select records related to tetramer assays for GAD, from the advanced search the Molecule Finder was used to select GAD and the Assay Finder to specify ‘tetramer.’ Figure 4B. Elements of query for T cell assay data specifying allele To search for peptides of a specific restriction as defined in vitro as potential tetramer targets the home page interface can be used to specify ‘T cell responses’ and the ‘MHC Class’ limited to class I (or specific allele of interest). Figure 4C. MHC binding data for GAD To search for peptides of a specific restriction as defined in binding assays, select ‘MHC binding data’ from the home page Immune Recognition Context section.

To select records related to tetramer assays from the advanced search, the Molecule Finder was used to select GAD and the Assay Finder to specify ‘tetramer.’ The results include 9 GAD epitopes for which tetramers were reported, from a total of 24 assays (Figure 4A). Restricting alleles include IAg7, H-2Kd and HLA-DRB1*04:04 in humans and NOD, NOR and TCR transgenic mice. In certain studies, epitopes have been modified to enhance MHC binding or for tetramer production. Therefore a query selecting analogs of a native sequence may reveal useful targets. For example, the GAD epitope NFFRMVISNPAAT, whereby the analog NFIRMVISNPAAT shows enhanced activity [24–26].

In another approach, a query was performed using the home page interface to specify ‘T cell responses’ and the ‘MHC Class’ was limited to class I, returning a total of 39 GAD epitopes derived from 22 references. Clicking on the ‘12 Restricting MHC Allele’ in the summary portion of the table gives the range of alleles/serotypes (Figure 4B). To “drill down” on a specific restriction element, the user may simply use ‘Revise Search’ from the results summary and the Allele Finder to specify the allele of interest, for example, ‘HLA-A2’.

Finally, to retrieve MHC binding data we selected “MHC Binding” as Immune Recognition Context. The GAD query returns a total of 193 peptides derived from 38 references. By clicking on the ‘453’ positive MHC binding assays we obtain a summary table of all records (multiple pages; Figure 4C), including assay description and binding affinity (ex. IARFKMFPEVKEK human GAD 2 (253–265), HLA-DRB1*04:05, Purified MHC Radioactivity Competition, IC50= 28nM). Of the 193 GAD epitopes tested, 25 records are associated with an IC50 value of 500nM or less. This query can also be revised using the Allele Finder to specify alleles or serotypes of interest.

Table 3 provides MHC restriction associated data for the top 5 auto antigens. A total of 34 epitopes have been defined by tetramer assays, including 20 class I alleles and 16 class II alleles. No tetramer data were available for IA-2 and ZnT8. To date, insulin has been more extensively studied than GAD or IGRP. In the other categories of T cell assays and MHC binding, GAD and insulin accounted for the majority of data, and class II epitopes outnumbered class I epitopes.

### Exploring diabetes associated data as a function of host organism or clinical status

**Figure 5:**
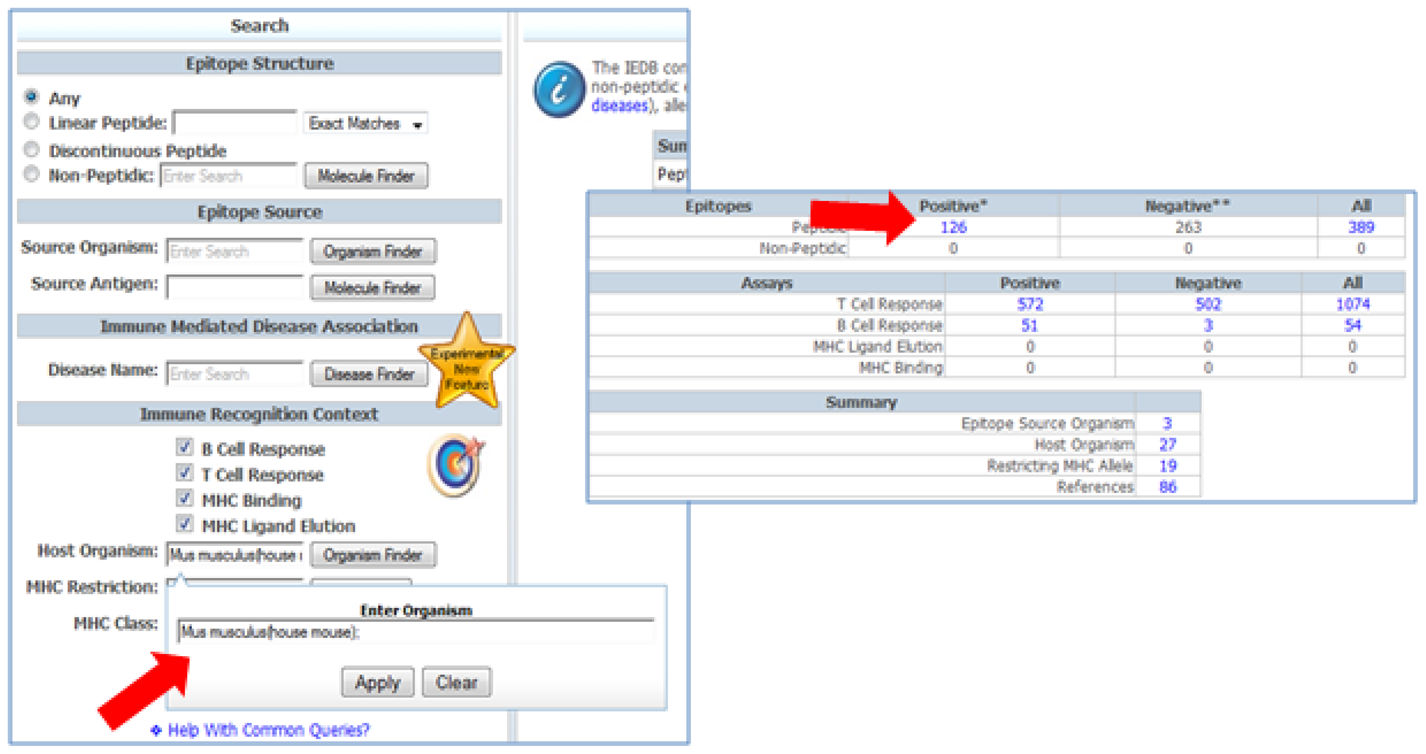
GAD epitopes defined in murine hosts To query for epitopes defined for GAD in mice, the Host Organism field was used to enter ‘Mouse.’ The auto-complete feature will display the top ten hits and by choosing ‘Mus musculus (ID 10090 common name: mouse’ the query will retrieve epitopes defined in mice for the specified antigen (in this example GAD).

Diabetes associated epitopes have been defined in humans and mice and to a lesser extent rats, rabbits, guinea pigs. It is often of interest to select epitope data relating to a specific host. For example, to query for epitopes defined for GAD in mice, the Host Organism field is used to enter ‘Mouse.’ The auto-complete feature will display the top ten hits and by choosing ‘Mus musculus (ID 10090 common name: mouse’ the query will retrieve epitopes defined in mice for the specified antigen (in this example GAD). This query retrieves a total of 126 epitopes reported in 86 references (Figure 5).

Table 4 provides host-specific data for the top 5 auto antigens from Table 1b. The number of epitopes is similar between human and murine hosts, for GAD and insulin. IA-2 and ZnT8 epitopes were predominantly defined in humans and IGRP epitopes primarily reported in mice (not shown).

**Figure 6:**
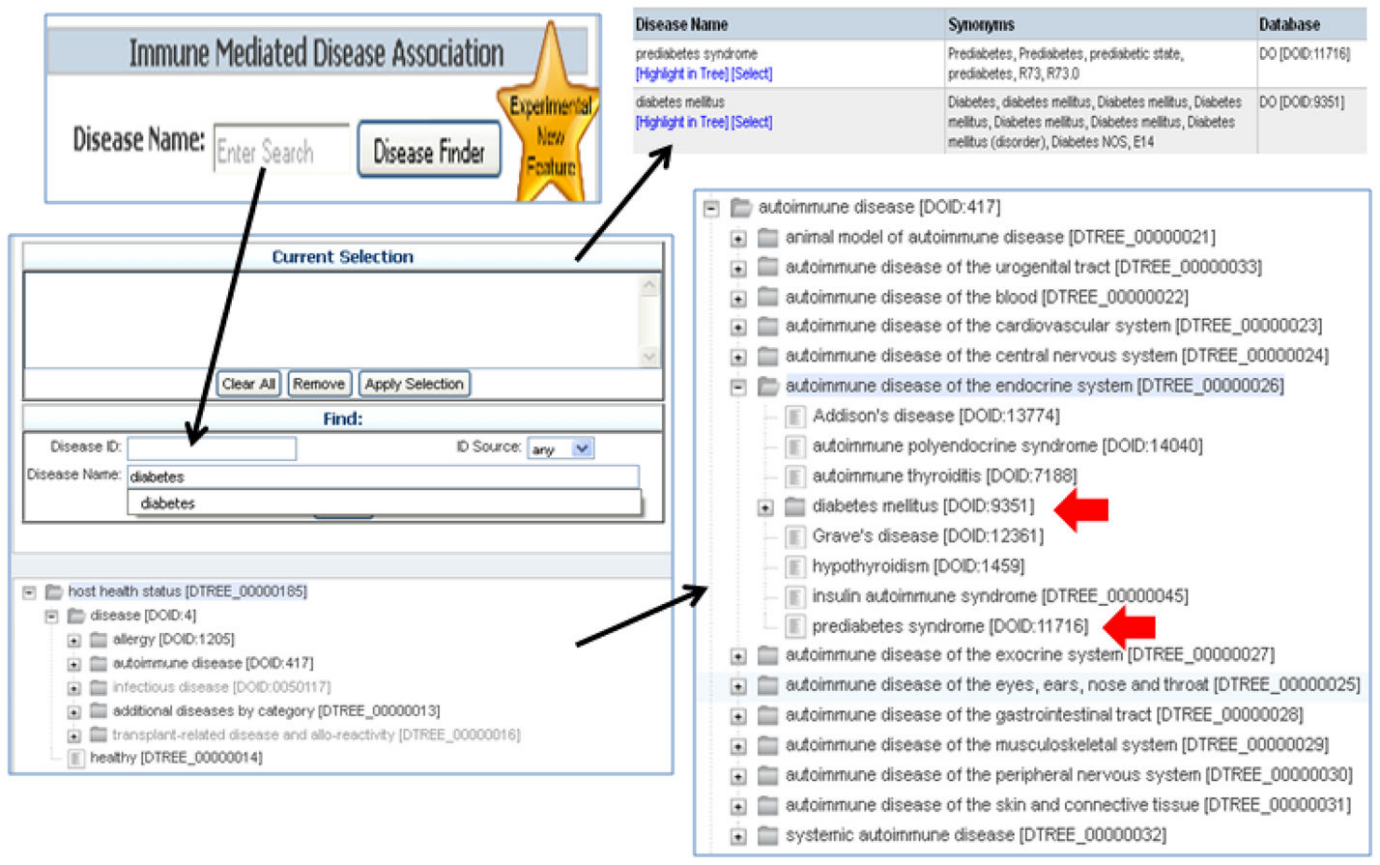
Disease Finder The Disease Finder feature selects data defined in hosts (both human and non-human) affected by a naturally-occurring or experimentally induced disease, excluding data derived from healthy controls and non-relevant data. The Disease Finder is accessible on the home page.

The Disease Finder feature selects data defined in hosts (both human and non-human) affected by a naturally-occurring or experimentally induced disease, excluding data derived from healthy controls and non-relevant data (e.g. MHC binding data). The Disease Finder is accessible on the home page and represents high level categories of disease, including ‘autoimmune disease.’ Entering, ‘diabetes’ into the Disease Name field generates a list of diseases captured to date that are related to diabetes. A Disease Tree is available and organized according to five broad categories, with each high level node further sub-categorized by anatomical location. The tree also includes healthy, controls [healthy, DTREE_00000014] (Figure 6).

Table 5 shows a query performed for ‘diabetes mellitus (DM)’ and ‘prediabetes.’ To date, 569 epitopes and 103 analogs from 211 references have been reported for DM, related to over 1,000 T cell assays and/or nearly 300 B cell assays. Prediabetes data was related to 271 epitopes and 105 analogs reported from 109 references. These epitope was also predominantly described in T cell assays. Of note, these data represent T1D. To date, a mere 6 references are captured in the IEDB describing T2D; however, this number is likely to increase in the coming years.

### Visualizing epitopes in the context of their antigen source

We recently developed an approach to visualize the results from multiple assays for a given antigen [27]. This ‘Immunomebrowser’ plots query results onto the specified antigen by calculating a response frequency score (RFscore) for each residue [27]. This feature is accessible from the Search Results Summary page by, clicking on ‘View In Immunome Browser.’

**Figure 7:**
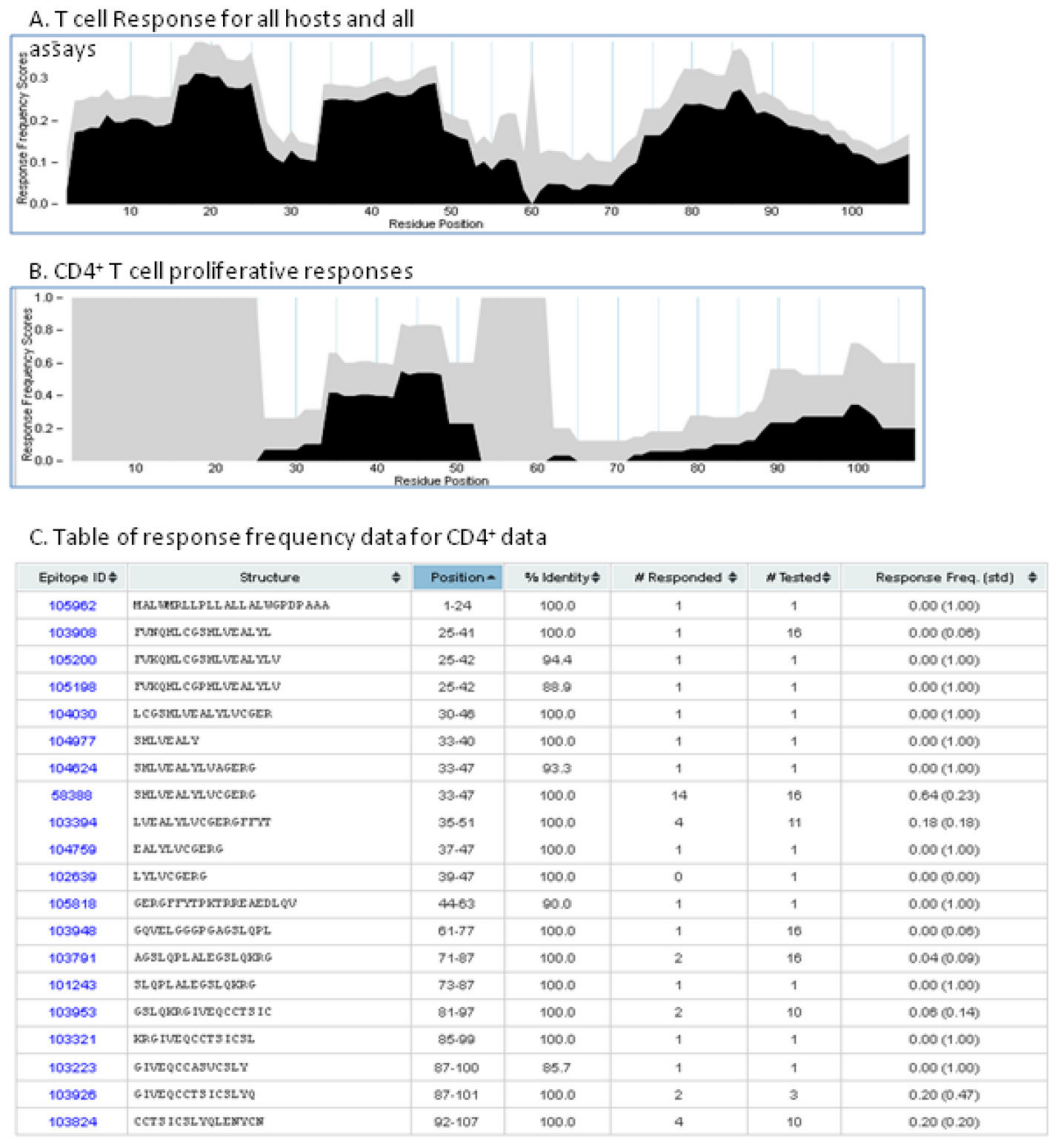
Immunobrowser: RFscores for all hosts mapped to human insulin Response frequency scores as a function of residue position are shown. A response frequency score is defined as: score = (responded-sqrt(responded))/tested. Variables ‘responded’ and ‘tested’ refer to number of individuals responded and tested, respectively. Black region indicates conservative estimates of response frequencies. Height of gray region indicates level of confidence associated with each response frequency score.

Figure 7A shows RFscores for T cell data for human insulin protein (GI: 1247492) as a reference antigen. While T cell reactivity has been described for essentially for the entire antigen, querying data related to CD4^+^ T cell epitopes defined for in lymphoproliferation assays (Figure 7B) reveals discrete regions found to be positive in 20–30% of those tested (RFscore ~0.20–0.30). These regions correspond to 22 different peptide epitope structures, some of which overlap sufficiently to be compatible with a single epitope. The summary table of RFscores for this query (Figure 7C) shows each overlapping register by position, its sequence and how many times it was tested. The search can be further refined by including additional criteria, such as a specific host species.

In a different application, we probed the IEDB for conformational B cell determinants. A query of B cell records associated with diabetes mellitus and prediabetes returned 232 positive B cell epitopes from 68 references. If the query is revised by changing the Epitope Structure’ type from ‘Any,’ (the default) to ‘Discontinuous Peptide,’ 26 discontinuous epitopes are found, described in 32 assays from 14 references (Table 6). These epitopes are derived from GAD, insulin, IA-2 and ZnT8 using antibodies from human subjects with disease.

**Figure 8:**
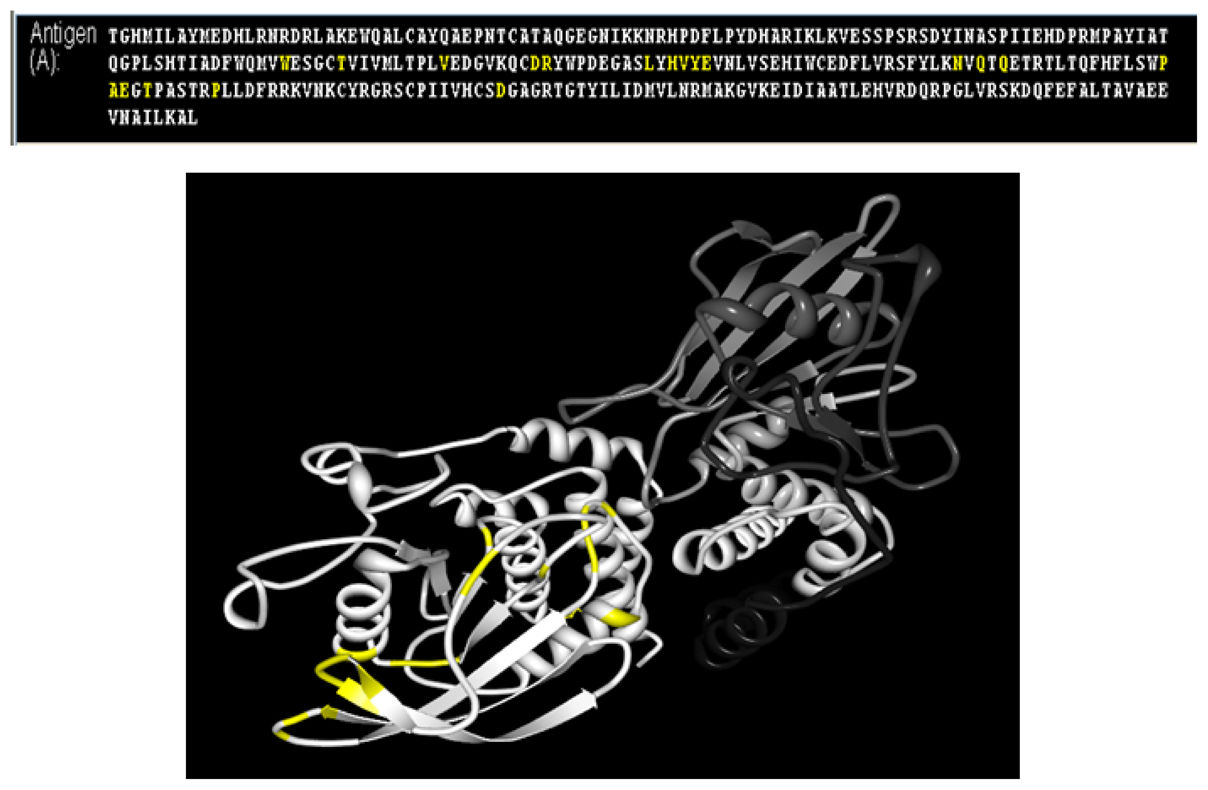
3D representations of IA-2 conformational epitopes These example 3D images were generated using the 3D Viewer function on the Homology Mapping tool for epitope all epitopes (in numerical order) defined for IA-2 antigens mapped to GI: 4506321. Epitope residues are shown in yellow.

The Homology Mapping tool visualizes conformational epitopes by mapping each residue onto a given antigen sequence and highlights its position in the 3D structure of the protein [28]. For example, a list of IA-2 epitopes was generated through Excel download. Next, from the ‘Tools’ menu we selected ‘Epitope Analysis Tools’ and then Homology Mapping. All IA-2 epitope residues reported (multiple records) were pasted in sequentially. The FASTA sequence of the GenBank ID (4506321) for IA-2 was then entered as source antigen, to generate the three-dimensional image shown in Figure 8.

## DISCUSSION

The goal of the present study is to raise awareness of the IEDB and its use within the scientific community devoted to autoimmune diseases. We show how the IEDB can be used to answer questions related to diabetes, including how to gather epitope data related to various diabetes-associated antigens, how to retrieve data related to a specific epitope, or epitopes restricted by a given allele. Additional questions include how to retrieve epitopes for which tetramers exist, how to visualize epitopes on the antigen’s three dimensional structure, and how to separate data derived from prediabetic versus diabetic individuals.

Herein we report >2,500 unique epitope structures defined in the context of T cell, B cell, MHC ligand elution and/or binding assays. The top diabetes-related antigens were GAD, insulin, IA-2/PTPRN, IGRP, ZnT8, HSP, and ICA-1, representing nearly 90% of the captured data. This distribution reflects antigens well-known to be associated with diabetes. However, the balance of type of epitopes varied amongst antigens. T cell assays were most numerous, except for IA-2 where there were a similar number of T and B cell assays. A relatively high number of papers related to heat shock proteins, possibly because of their association with molecular mimicry. However qualifying hsp60 as a target in T1D autoimmunity is debatable [12–14, 29]. Also unexpected was the relatively low number of epitopes related to chromogranin-A, recently identified as a diabetes-related auto antigen, exclusively in the NOD mouse (10). These results illustrate potential data gaps for some antigens and differences in the nature of defined epitopes, thus highlighting potential areas for further research.

Genetic predisposition to T1D is associated with MHC class II loci, DQA1, DQB1, and DRB1 in humans and IAg7 in NOD mice [17–23]. MHC restriction data is important to gain a better understanding of the disease processes and to develop tools for diagnosis [15, 16]. Herein we described three strategies for searching for these data. Restricting queries to a particular host revealed that human data represent the largest portion, followed by mice (mostly NOD) and with far fewer data reported from other species. Interestingly, while the epitope distribution is fairly similar between human and murine hosts, for GAD and insulin, epitopes defined for IA-2 have been predominantly defined in humans, those for IGRP were primarily reported in mice and those for ZnT8 were exclusively defined in humans.

The IEDB was originally focused on infectious diseases, and as such the expansion of its focus to other areas of immunological interest such as allergy [30] and autoimmune diseases [31] is dictating the development of additional and novel query and reporting tools. While in the context of infectious diseases, the relevant records can be easily obtained by searching for the etiologic agent (e.g. Influenza), this is not possible for autoimmune diseases, where most studies focus on a few specific antigens. The protein tree was developed to allow selection of records associated to a given antigen. The prototype version of this tool is already deployed in the IEDB and it is being further enhanced.

The Disease Finder is another recently developed feature of the IEDB that allows querying records related to a given disease, or healthy control subjects. For example, comparisons can be made between records describing T and B cell epitopes defined in diabetic subjects and prediabetic subjects. While there is no pathognomonic epitope or antigen identified to date, many studies show significant differences between groups. Furthermore, our analysis showed a paucity of conformational B cell epitope data, thus highlighting an important area for further study.

Despite the overall lack of broad conformational epitope coverage, the existing data represent the major auto antigens of diabetes in humans, namely GAD, insulin, IA-2 and ZnT8.

Finally, it is important to consider the issue of investigational bias. The global collection of data from the literature can reflect investigational biases present in primary publications. In the case of diabetes, potential biases relate to the preferential identification of T cell epitopes from auto-antibody targets versus a more broad investigation or the experimental modification of epitopes to improved HLA binding, resulting in potentially flawed tetramer measurements. Furthermore, some investigations a priori assumed that peptides must have high affinity binding to bind to MHC, while recent studies suggest that this may be more exception than rule in case of autoimmunity [3, 32, 33].

It is important to note that the IEDB cannot solve the fundamental issue of investigational bias in terms of how the scientific community designs, executes and report studies. Indeed, we typically find that different users disagree on what they consider relevant, or conversely, biased. While the IEDB cannot alter or exclude data, or directly address and prevent investigational bias, our goal is to provide the user with a sophisticated platform upon which to perform queries tailored to their preferences and capable of excluding data they do not deem appropriate. This type of approach has the additional benefit of revealing areas of significant gaps in the knowledge base and therefore highlights targets for future investigation.

The diabetes related data contained in the IEDB can be seamlessly linked to the IEDB analysis resource, [34], which includes class I, class II and B cell epitope prediction tools, and other epitope analysis tools such epitope clustering, homology mapping, conservancy analysis and population coverage tools. Moreover, the IEDB provides links to more than 50 other relevant databases/resources, including NCBI, GenBank, PDB, ChEBI, DO, etc. This combination of data, features and tools housed in one location makes the IEDB unique among immunological web-based resources.

Herein we demonstrate the use of the IEDB to access and analyze diabetes-specific epitope data generated from humans and animal models. Our ultimate objective is to increase the awareness of the IEDB resource in the diabetes research community, and encourage feedback, which is key to ensuring the accuracy and continued enhancement of the data housed therein.

## METHODS

### Data inclusion criteria

This analysis includes available data for antibody and T cell epitopes associated with diabetes, based either on the clinical status of the host or on the association of the antigen with diabetes. We followed the process of Davies et al [7] to identify diabetes related data derived from peer-reviewed literature (PubMed), as well as data directly submitted to the IEDB (instructions can be obtained on the IEDB main page Solutions Center under ‘Data Submissions.’). Epitope definitions (length and mass restrictions) and IEDB inclusion criteria can be found at [http://tools.immuneepitope.org/wiki/index.php/Curation_Manual2.0#Prevailing_Rules]. For the purpose of this report, epitopes represents the unique molecular structures (minimal sequences, linear and discontinuous regions, as well as key residues) experimentally shown to react with a B cell or T cell receptor (the database only curates experimental data and does not include predictions).

### IEDB Queries and Analysis

Queries were performed using the IEDB [www.iedb.org] search interfaces. Unless otherwise specified, results were analyzed by downloading them into Excel format. In some cases, query results were captured as still images. All other figures and/or tables were produced in Excel. The Response frequency score (RFscore) is calculated as described previously [27].

## Figures and Tables

**Table 1:** Diabetes-related data based on IEDB classification While the Davies classification (7) is not currently accessible from the IEDB webpage, these results can be approximated by performing a keyword search of the IEDB for the word ‘diabetes’ which produces a total of 2,624 epitopes and 1,651 negative structures, reported from 432 references. This search function is approximate, and should only be considered as a general indicator of reference numbers. Disease-association query strategies currently in development (see below) will allow assembling diabetes related.

Total number of structures	**5,051**
Positive structures (epitopes)	**2,537**
Negative structures	**2,514**
Total references	**409**

**Table 2:** Top 15 diabetes-associated auto antigens Top 15 antigens were chosen based on occurrences and/or number of epitopes defined therein, and include only those with at least 2 occurrences.

	Auto antigen	T assays	B Assays	Elution	MHC	Total Assays	Ag/ref
1.	GAD	947	195	2	514	1,658	209
2.	Insulin	577	71	7	256	911	193
3.	IA-2/PTPRN	137	107	35	144	423	41
4.	IGRP/G6Pase	285	0	1	19	305	34
5.	ZnT8	152	3	0	66	221	6
6.	HSP	145	52	2	14	213	40
7.	ICA-1	12	0	0	136	148	7
8.	MHC	40	21	5	20	86	29
9.	GFAP	45	0	0	5	50	6
10.	IGF	0	43	0	0	43	2
11.	Serum albumin	34	4	0	3	41	12
12.	IAPP	8	11	0	5	25	7
13.	TCR	14	3	0	6	23	9
14	LPS/LOS	20	0	0	0	20	4
15	Chromogranin-A	12	0	0	6	18	2

Abbreviations: GAD, glutamate decarboxylase; HSP, heat shock protein (chaperonin); IA-2, insulinoma antigen 2; PTPRN, protein tyrosine phosphatase, receptor type, N and receptor-type tyrosine-protein phosphatase-like N; G6pase, glucose-6-phosphatase, including islet-specific G6Pase-like protein; MHC, major histocompatibility complex; TCR, T cell receptor; ICA-1, islet cell auto antigen 1; IAPP, islet amyloid polypeptide precursor; ZnT8, zinc transporter 8; IA2. Islet auto-antigen 2 (protein tyrosine phosphatase-like auto-antigen 2); IGF, insulin-like growth factor 1; LPS/LOS, lipopolysaccharide/lipooligosaccharides; GFAP, glial fibrillary acidic protein isoform 2. A total of 405 references were identified [using method described in Davies 2009] as being associated with ‘diabetes’ as of September 2012. Enumeration of total assays: these data were generated from the SQL download. A spreadsheet of all data (all ref IDs = diabetes from Davies 2009) was first filtered for ‘qualitative_measure’ = positive. Then using the ‘structure_source_ag’ field each individual auto antigen was selected and the total number of ‘struc_id’s (unique epitopes) were enumerated using advanced filter to retrieve only unique IDs. Then Bcell_id, Tcell_id, MHC_id and elution_ids were enumerated. The total number of positive assays for auto antigens with ‘mammal’ as source species = 4,433.

**Table 3:** Broad inventory of restriction associated data for top 5 auto antigens

	Antigen Name	Epitopes	Tota1 Assays	Class I alleles	Class II alleles	References
	GAD	9	24	2	7	10
Tetramer*	insulin	18	35	16	3	14
	IGRP	7	21	2	6	10
		**34**	**80**	**20**	**16**	34
	GAD	141	625	34	115	94
T cell Assays	insulin	97	416	42	57	77
With	IA-2	39	80	8	31	12
Restriction	IGRP	75	281	63	12	26
	ZnT8	20	60	18	2	4
		**372**	**1,462**	**165**	**217**	**213**
	GAD	182	453	20	162	38
	insulin	142	352	92	52	40
MHC Binding	IA-2	78	154	18	60	11
	IGRP	12	19	12	0	8
	ZnT8	62	66	31	31	3
		**476**	**1,044**	**173**	**305**	**100**
**Totals**		**882**	**2,586**	**358**	**538**	**347**

* Data were not available for IA-2 and ZnT8. T cell assays retrieved for top antigens having any defined restriction; class I/II, allele undetermined not included.

**Table 4:** Breakdown of host species for top 5 auto antigens

Host	Epitopes	T cell Assays	B cell Assays	Ligand Elution	References
**Human**	365	766	113	45	139
**Mouse**	311	1194	86	1	170
**Rabbit**	31	0	107	0	12
**Rat**	4	16	0	0	2
**Guinea pig**	1	1	0	0	1

Top 5 antigens include GAD, insulin, IA-2, IGRP, and ZnT8. Hosts are reported as a group; however, they include multiple mouse strains, including HLA-transgenic and humans from certain geographical locations. All data = ‘+’

**Table 5:** Retrieving epitopes in the context of disease

Assay Type	Epitopes	Analogs	T cell Assays	B cell Assays	Elution	Reference
**Diabetes mellitus**	569*	103	1013	278	1	211
**Prediabetes**	271**	105	795	44	0	109

These data include all antigens and all hosts.

* Includes 17 non-peptidic structures;

** includes 5 non-peptidic structures. Analogs were enumerated by Excel download of epitope list. ‘Diabetes mellitus’ was chosen versus ‘insulin-dependent diabetes mellitus (IDDM)’ because the majority of papers report subjects as ‘diabetic’ and do not specify IDDM, per se. Only 6 epitopes were defined as non-IDDM.

**Table 6:** Conformational epitopes defined in the context of clinical disease

Epitope Sequence	Antigen	Source
E517	GAD 2	Human
R255, F256, K257, K263, E264, K265, L270, P271, R272, L273, L285, K286, K287, I294, G295, T296, D297, S298, R317, R318	GAD 2	Human
N483, I484, I485, K486, N487, R488, E489, G490, Y491, E492, M493, V494, F495, D496, G497, K498, P499, F556, F557, R558, M559, V560, I561, S562, N563, P564, A565, A566, T567, H568, Q569, D570, I571, D572, F573, L574, I575, E576, E577, I578, E579, R580, L581, G582, Q583, D584, L585	GAD 2	Human
N483, I484, I485, K486, N487, R488, E489, G490, Y491, E492, M493, V494, F495, D496, G497, K498, P499, F556, F557, R558, M559, V560, I561, S562, N563, P564, A565, A566, T567, H568, Q569, D570, I571, D572, F573, L574, I575, E576, E577, I578, E579, R580, L581, G582, Q583, D584, L585	GAD 2	Human
E264	GAD 2	Human
E517, E520, E521, S524, S527, V532	GAD 2	Human
E517, E521	GAD 2	Human
K358	GAD 2	Human
R536, Y540	GAD 2	Human
F25, V26, N27, E37, R46, T51, T85, S86, I87, S89, L90, Y91, Q92, E94	insulin	Human
P52, K53, L90	insulin	Human
F25, V26, N27, T97, S98, I99, C100, S101, L102	Insulin	Human
F25, V26, N27, E37, R46, T51, P52, K53, T54, T85, S86, I87, S89, L90, Y91, Q92, E94	insulin	Human
F25, V26, N27, E37, R46, T51, T54, T85, S86, I87, S89, L90, Y91, Q92, E94	insulin	Human
P876, A877, E878, T880	IA-2	Human
T804	IA-2	Human
T804, V813, D821, R822, Q862, P886	IA-2	Human
W799, E836, N858	IA-2	Human
D911	IA-2	Human
Q862	IA-2	Human
L831, H833, V834, E836, Q860	IA-2	Human
W799, E836, N858	IA-2	Human
W799, L831, H833, V834, Y835, E836, Q860	IA-2	Human
R325, R332, E333, K336, K340	ZnT8	Human
R325	ZnT8	Human
W325	ZnT8	Human
